# The reticulon protein, TtRET1, is required for the initiation of mating in *Tetrahymena thermophila*

**DOI:** 10.17912/micropub.biology.001763

**Published:** 2025-08-08

**Authors:** Sabrice Guerrier, Jordon Williams, Brandon Garcia

**Affiliations:** 1 Biology, Rollins College, Winter Park, Florida, United States

## Abstract

We recently identified *Tetrahymena thermophila* reticulon (TtRET1) as a novel marker of the endoplasmic reticulum (ER) that reveals ER morphology during the conjugation phase of mating, but its functional role was previously unknown. Here, we show that TtRET1 is required for the early initiation of mating, prior to conjugation. Furthermore, TtRET1 relocalizes during the mating reaction, suggesting it may regulate ER remodeling events necessary to initiate the mating program.

**Figure 1. TtRET1 is required for pair formation and costimulation in T. thermophila mating:**
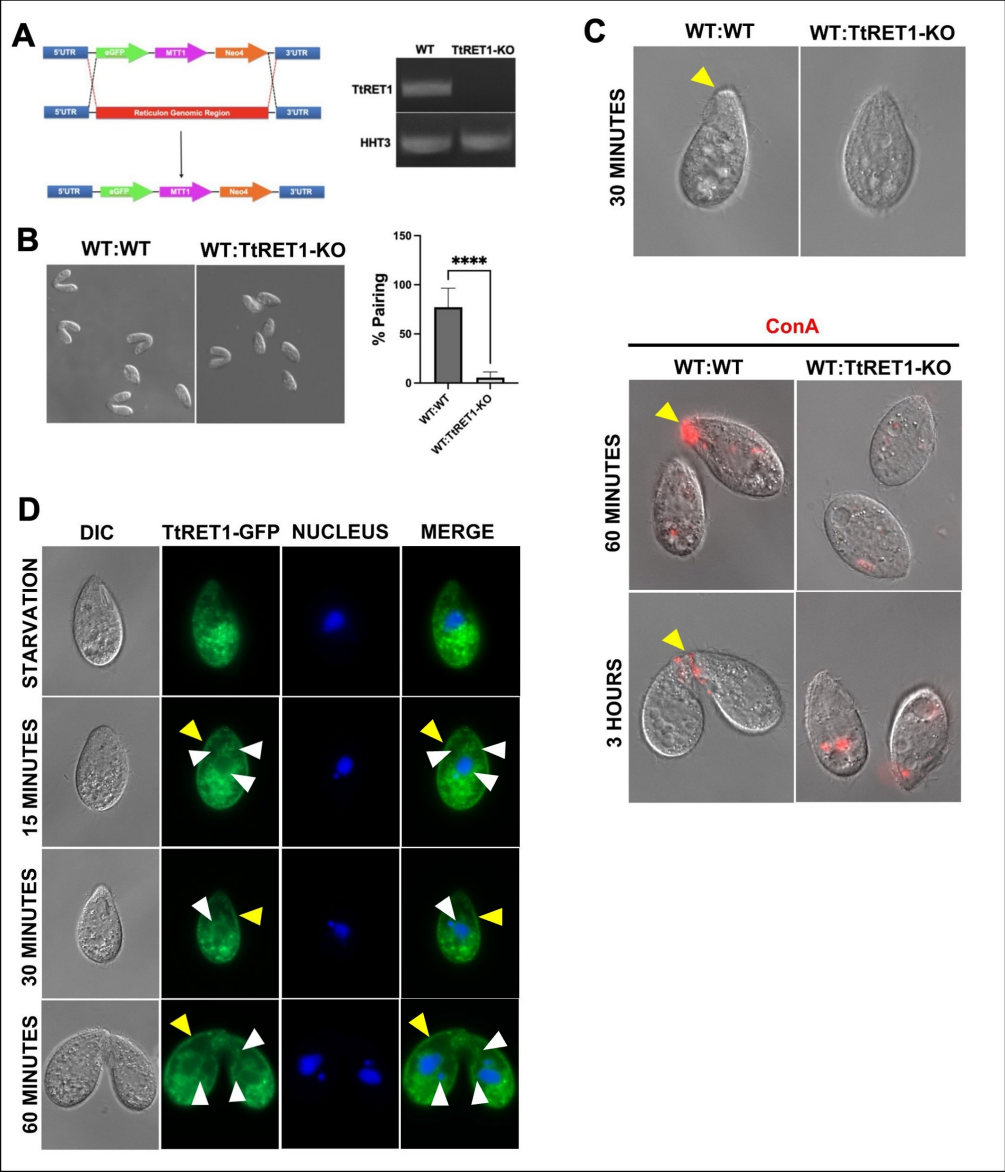
**A.** Schematic of the *TtRET1* gene knockout strategy. The endogenous *TtRET1* locus was replaced with the NEO4 cassette via homologous recombination to generate *TtRET1* knockout (TtRET1-KO) cells. Right: RT-PCR showing *TtRET1* expression in wild-type (WT) and TtRET1-KO cells. Histone H3 ( *HHT3* ) served as a control for cDNA quality. **B.** Micrographs showing pairing in WT × WT and WT × TtRET1-KO matings. Right: Quantification of pairing frequency (N = 3 experiments; over 100 cells counted per experiment). Data were analyzed using the Kruskal–Wallis test; error bars represent standard deviation. **** indicates p < 0.0001. **C.** Top: Micrographs of WT × WT and WT × TtRET1-KO cultures incubated for 30 minutes. Yellow arrowheads indicate tip transformation. Bottom: Cells incubated for 60 minutes or 3 hours, labeled with ConA-Rhodamine (Vector Laboratories). Yellow arrowheads indicate ConA enrichment at the anterior. **D.** Localization of TtRET1-GFP in starved cells and during mating (15, 30, and 60 minutes after mixing with WT cells). White arrows represent pericunuclear and tubular localization while yellow indicates cortical localization.

## Description

Sexual reproduction/mating in the ciliate *Tetrahymena thermophila* ( *T. thermophila* ) involves multiple stages: partner recognition (costimulation), pair formation (physical association facilitated via adhesion followed by membrane fusion), and conjugation (nuclear exchange) (Cole, 2007). Previous studies suggest that the endoplasmic reticulum (ER) extends from the perinuclear region to the plasma membrane within the site of nuclear exchange (i.e. conjugation junction) in *T. thermophila* (Cole et. al., 2015; Guerrier et al., 2024). In our recent study, we found that both *T. thermophila* reticulon TtRET1 and the ER lumen marker KDEL localized similarly to the perinuclear region and to tubular extensions that contact the plasma membrane (Guerrier et al., 2024). These observations, combined with the established role of reticulon proteins as ER membrane tubulators, identify TtRET1 as a novel ER marker in *T. thermophila* . We hypothesized that ER tubulation could promote ER–plasma membrane contact sites that support lipid exchange, since membrane fusion during conjugation is associated with a shift from curvature-resisting to curvature-accommodating lipids (Ostrowski et al., 2004; Wong et al., 2019). However, the role of the ER in *T. thermophila* conjugation has not previously been tested.

To address this, we generated *TtRET1* knockout (KO) cells by replacing the *TtRET1* gene with a green fluorescent protein (GFP) and the NEO4 cassette ( Figure 1A ). RT-PCR confirmed the absence of *TtRET1* expression in the knockout strain ( Figure 1A, right). When mixed with wild-type (WT) cells, *TtRET1* -KO cells showed significantly reduced pairing compared to WT:WT matings after three hours ( Figure 1B ). This extremely low level of pairing suggested that mating partners had difficulty in recognition.

To assess early events in the mating program, we examined markers of costimulation, including tip transformation and Concanavalin A (ConA) labeling. WT: *TtRET1* -KO matings failed to exhibit either marker ( Figure 1C ), indicating that *TtRET1* is required for costimulation. This supports the idea that ER remodeling may be an early component of the mating response.

To explore this further, we examined TtRET1-GFP localization in starved and mating cells. In starved cells, TtRET1-GFP displayed diffuse localization ( Figure 1D ). Upon mixing with mating partners, its localization shifted to the cortical ER (yellow arrowheads), perinuclear ER and tubular structures (white arrowheads) ( Figure 1D ). A similar pattern was observed in KDEL-GFP expressing cells (Guerrier et al., 2024), further supporting that TtRET1 localizes to the ER. These findings suggest that ER reorganization is part of the early mating program in *Tetrahymena* .

The phenotype of *TtRET1* -KO cells resembles that of ΔMTA/ΔMTB double knockout cells, which lack the mating type receptors MTA and MTB (Yan et al., 2024). This raises the possibility that TtRET1 facilitates MTA/MTB receptor localization, consistent with the role of reticulon-mediated ER tubulation in receptor trafficking in other systems (Lee et al., 2011). Alternatively, TtRET1 may act downstream of MTA and MTB, promoting the membrane changes necessary for tip transformation and ConA receptor exposure. Notably, CDK19, reported to interact with MTA and MTB (Yan et al., 2024), also leads to costimulation defects when deleted (Ma et al., 2020). Cyclin-dependent kinases have been shown to regulate ER structure during mitosis (Bergman et al., 2015; Wang et al., 2013), and the localization of ER-shaping proteins, like reticulon, can be modulated by phosphorylation (Jiang et al., 2020; Schlaitz et al., 2013). These findings establish TtRET1 as a regulator of conjugation initiation and highlight the changes in ER morphology as a likely part of the initiation program.

## Methods

***Generation of TtRET1 knockout Tetrahymena***

*TtRET1* 5’untranslated region along with open reading frame (1533bp) and 3’ untranslated region (1075bp) were amplified and subcloned into NHEI and SALI sites, respectively that flank the neo4 cassette, using infusion cloning (Takara inc.). This construct resulted in deletion of the entire open reading frame of TtRET1. The resulting vector was linearized and introduced into Tetrahymena (both CU427 and B2086 cell lines) using biolistic transformation (Cassidy-Hanley et al., 1997). TtRET1-KO Tetrahymena were selected by growth in SPP media and increasing concentrations of paromomycin. To assess gene disruption, we isolated total RNA using Trizol followed by RNA cleanup (Qiagen). The presence of TtRET1 transcripts was then assessed using SuperScript™ IV First-Strand Synthesis System with ezDNase (Fisher Scientific) Enzyme for cDNA synthesis followed by PCR.

***Tetrahymena mating reactions***

All incubations take place at 30 ^o^ C. TtRET1-KO (B2086 minus TtRET1), Wild Type (WT) Tetrahymena (CU427, B2086 or CU427, B2086 cells expressing TtRET1-GFP) were grown overnight to 200,000 cells per ml. Cells were then washed and starved in 10mM Tris pH 7.4 overnight. For pairing and costimulation assays, starved TtRET1-KO and TtRET1-GFP expressing CU427 Tetrahymena (WT:TtRET1-KO matings) or CU427 and B2086 Tetrahymena expressing TtRET1-GFP (WT:WT matings) were mixed at equal concentrations. When indicated, mating reactions were then treated with NucBlue (Thermofisher) nuclear stain. Mating reactions were then immobilized using NiCl and imaged immediately using Zeiss Axio Observer with Apotome.

***Assessment of Pairing***

Mating cultures were prepared as described above. To assess pairing, mating cultures were incubated for 3 hours then immobilized using NiCl and imaged. The percentage of pairs was calculated by counting at least 100 objects (single cell or pair) in at least 10 fields of view. The percentage of pairing was calculated as (number of single cells or pairs/total number of objects) x 100. This data was plotted and analyzed using Graphpad Prism.

***Costimulation assessment***

Mating cultures were prepared as described above. Differential Interference Contrast (DIC) images were used to assess tip transformation (elongation or flattening of the anterior portion of the cell). For Concanavalin (ConA-Rhodamine (Vector Laboratories) staining, 1ml of mating culture was extracted (at 15, 30, 60 minutes and 3 hours) then incubated with 50ug/ml of ConA-Rhodamine for five minutes at 30 ^o^ C. This was subsequently centrifuged then washed with 10mM Tris three times. ConA-Rhodamine treated mating reactions were then immobilized using NiCl and imaged immediately using Zeiss Axio Observer with Apotome.
